# Cortical Thickness Estimations of FreeSurfer and the CAT12 Toolbox in Patients with Alzheimer's Disease and Healthy Controls

**DOI:** 10.1111/jon.12521

**Published:** 2018-05-15

**Authors:** Rene Seiger, Sebastian Ganger, Georg S. Kranz, Andreas Hahn, Rupert Lanzenberger

**Affiliations:** ^1^ Department of Psychiatry and Psychotherapy Medical University of Vienna Vienna Austria; ^2^ The State Key Laboratory of Brain and Cognitive Sciences The University of Hong Kong Pokfulam Hong Kong; ^3^ Laboratory of Neuropsychology The University of Hong Kong Pokfulam Hong Kong

**Keywords:** Cortical thickness, FreeSurfer, CAT12, SPM, Alzheimer's disease

## Abstract

**BACKGROUND AND PURPOSE:**

Automated cortical thickness (CT) measurements are often used to assess gray matter changes in the healthy and diseased human brain. The FreeSurfer software is frequently applied for this type of analysis. The computational anatomy toolbox (CAT12) for SPM, which offers a fast and easy‐to‐use alternative approach, was recently made available.

**METHODS:**

In this study, we compared region of interest (ROI)‐wise CT estimations of the surface‐based FreeSurfer 6 (FS6) software and the volume‐based CAT12 toolbox for SPM using 44 elderly healthy female control subjects (HC). In addition, these 44 HCs from the cross‐sectional analysis and 34 age‐ and sex‐matched patients with Alzheimer's disease (AD) were used to assess the potential of detecting group differences for each method. Finally, a test‐retest analysis was conducted using 19 HC subjects. All data were taken from the OASIS database and MRI scans were recorded at 1.5 Tesla.

**RESULTS:**

A strong correlation was observed between both methods in terms of ROI mean CT estimates (*R* ^2^ = .83). However, CAT12 delivered significantly higher CT estimations in 32 of the 34 ROIs, indicating a systematic difference between both approaches. Furthermore, both methods were able to reliably detect atrophic brain areas in AD subjects, with the highest decreases in temporal areas. Finally, FS6 as well as CAT12 showed excellent test‐retest variability scores.

**CONCLUSION:**

Although CT estimations were systematically higher for CAT12, this study provides evidence that this new toolbox delivers accurate and robust CT estimates and can be considered a fast and reliable alternative to FreeSurfer.

## Introduction

The cerebral cortex of the human brain is highly folded with an average thickness of around 2.5 mm, which varies between 1 and 4.5 mm across different brain regions.^1^ The analysis of cortical thickness (CT) allows for acquisition of valuable *in vivo* information about normal and abnormal neuroanatomy in the healthy and diseased human brain. This is of particular interest when participants in whom cognitive decline, or even dementia, such as Alzheimer's disease (AD), are investigated. AD is a neurodegenerative disorder, characterized by accumulation of beta‐amyloid and tau proteins, which are associated with neurodegeneration in the form of synaptic, neuronal, and axonal deterioration affecting memory and cognitive function.^2^ Neurodegeneration in AD typically begins in temporal lobe regions before affecting the neocortex.^3^ These atrophic patterns can be observed with structural magnetic resonance imaging (MRI) methods.^4^ For example, neuropathological studies revealed that especially temporal brain structures such as the entorhinal cortex, the parahippocampal gyrus, and regions around the superior temporal sulcus are affected by neurodegeneration in AD.^5^, ^6^ To assess these brain alterations in the form of brain atrophy, methods are needed that deliver reliable CT estimations.

**Figure 1:**
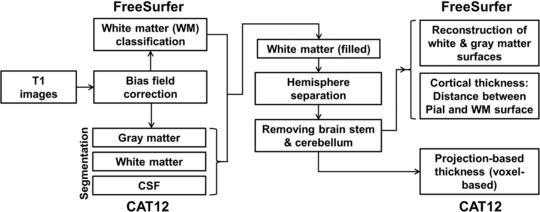
Flow‐chart comprising both methodological approaches.

Several methods for CT estimations have already been introduced^7^ and can be broadly classified as either surface‐based or volume‐based.^8^ While volume‐based methods share the advantage of lower processing times, surface‐based approaches excel in terms of accuracy, as the entire surface is modeled. FreeSurfer is an established software suite utilizing the surface‐based approach and can be considered the gold standard in CT measurements. It is frequently used for automated CT estimation with high accuracy^9^ where the entire cortical surface is reconstructed (see Fig 1). More specifically, a reconstruction step is performed in which the outer boundary, based on the inner boundary, is reconstructed through model‐based deformation of the inner surface.^1^, ^10^ Although the method delivers accurate thickness estimations, extensive processing times are inevitable. However, for some research questions, these extensive surface reconstruction steps are not necessary.

Recently, the computational anatomy toolbox (CAT12: http://www.neuro.uni-jena.de/cat/) for SPM (Statistical Parametric Mapping software, http://www.fil.ion.ucl.ac.uk/spm/) has been introduced, offering a fast and easy‐to‐use alternative approach for CT estimations without the extensive reconstruction of the surface. This volume‐based approach uses projection‐based thickness (PBT),^11^ where a projection scheme is used, using the information of blurred sulci to create a correct CT map. With the voxel‐based thickness, the estimation of PBT enables the easy creation of the central surface, which is thought to have better properties than the white matter (WM) or pial surface. This central surface is generated at the 50% distance boundary between gray matter (GM)/WM and GM/cerebral spinal fluid (CSF).^11^ It is now possible to include the estimation of the CT and the central surface of both hemispheres, based on the PBT method.^11^ The reconstruction of the surface includes topology correction, which accounts for topological defects using spherical harmonics.^12^ Furthermore, spherical mapping is applied to reparameterize the surface mesh into a common coordinate system,^13^ while spherical registration adapts the volume‐based diffeomorphic DARTEL algorithm^14^ to the surface.

In summary, this new PBT method leads to a tremendous reduction in processing time as no extensive reconstruction of the surface is performed. Furthermore, a graphical user interface as integrated in SPM simplifies the process for users not familiar with the command line. However, it remains to be investigated if both methods deliver comparable results. Here, we assessed CT estimations using both methods by comparing an AD cohort to healthy controls (HCs).

MRI data from HCs and from an AD cohort were processed with both CT approaches and a region of interest (ROI)‐wise comparison was carried out. First, we evaluated general differences in CT estimations for both methods and each ROI. Afterward, CT was directly compared between AD patients and HCs to assess the applicability in clinical research. In a last step, a test‐retest analysis was conducted in order to gain insights into the reliability of both methods.

## Methods

### Subjects

All data in this study were taken from the freely available Open Access Series of Imaging Studies (OASIS) database (http://www.oasis-brains.org/).^15^ For the cross‐sectional comparison between FreeSurfer 6 (FS6) and the CAT12 toolbox, 44 elderly healthy female subjects (77.8 ± 8.3 [mean age ± SD]) were included. Subsequently, the 44 HCs from the cross‐sectional analysis and 34 age‐ and sex‐matched AD patients (78.1 ± 7.7) (Mini‐Mental State Examination ≤ 26; Clinical Dementia Rating ≥ .5) were used to assess the potential for detecting group differences for each method. Furthermore, a test‐retest analysis was conducted using 19 HC subjects (23.6 ± 4.1, 11 females) from the OASIS reliability database, which were measured at two time points (days between scans: 21.5 ± 24.1). One subject out of the 20 available datasets was excluded prior to the final analysis due to processing issues.

### Data Acquisition

Structural MRI scans were acquired at a field strength of 1.5 Tesla with a T1‐weighted magnetization‐prepared rapid gradient‐echo (MPRAGE) sequence (voxel size = 1 × 1 × 1.25; dim = 256 × 256 × 128; TR = 9.7 milliseconds; TE = 4.0 milliseconds) utilizing a Siemens Vision scanner (Erlangen, Germany). For each subject, three to four individual images were acquired in a single session and averaged before further processing. To reduce motion artifacts, head positioning cushions and a thermoplastic face mask were applied. More details regarding the data acquisition can be found at Marcus et al.^15^

### Data Processing

#### FreeSurfer

The subjects were processed with the FreeSurfer software (http://surfer.nmr.mgh.harvard.edu/, version 6.0) using the “recon‐all” processing stream with default parameters to create a 3‐dimensional cortical surface model.^1^ After automated Talairach transformation^16^ and intensity normalization,^17^ nonbrain tissue was removed.^18^ Hemispheres were separated and cerebellum and brain stem were excluded. This was followed by a tessellation of the gray and WM boundary and topology correction.^19^ Furthermore, surface deformation enables the detection of tissue boundaries, while CT is calculated as the distance between the white and pial surface.^1^ For the longitudinal analysis, subjects were processed with the respective processing stream implemented in FreeSurfer. An unbiased within‐subject template was created^20^ using robust, inverse consistent registration.^21^ The Talairach transforms, atlas registration, spherical surface maps, and parcellations were initialized with the information from the prior generated within‐subject template.^20^ After the automated reconstruction of all subjects, volumes were visually inspected for misclassifications during the reconstruction process.

#### CAT12

In addition, all participants were processed with the CAT12 toolbox (http://www.neuro.uni-jena.de/cat/, version r1109) within SPM12 (http://www.fil.ion.ucl.ac.uk/spm/software/spm12/, version 6225) using MATLAB (8.3) to gather CT estimates. Initially, volumes were segmented using surface and thickness estimation for ROI analysis in the writing options. Estimation of CT and the central surface was performed in one step, based on the PBT method.^11^ Here, topology correction,^12^ spherical mapping,^13^ and spherical registration were carried out. After tissue segmentation, WM distance was estimated and the local maxima were projected to other GM voxels using a neighbor relationship described by the WM distance.^11^ The longitudinal data were processed using the longitudinal preprocessing option, where spatial normalization parameters are calculated using an average image of the two time points. This was again applied to the first and the second images. Hence, an additional registration step between the two images was introduced. Finally, data were visually inspected.

### ROI Extraction

For both methodological approaches, the mean CT values were extracted for 34 ROIs defined by the Desikan‐Killiany atlas^22^ using standard procedures for ROI extraction provided in both software suites. The estimated mean CT values for each ROI were then averaged between hemispheres as no lateralization effects were expected. Subsequent ROI data for the statistical models were transferred to SPSS (IBM SPSS Statistics 24).

### Statistics

#### Comparison of FS6 and CAT12 Using a Healthy Control Population

A linear regression model was calculated for the HCs to assess the agreement between FS6 and CAT12 CT ROI estimates. The mean CT values for all 34 regions for both methods were used for the analysis and coefficient of determination (*R* ^2^), slope, and intercept were calculated.

To compare both methods, a linear mixed model analysis was conducted to account for differences in CT estimations between the two approaches. Method (FS6, CAT12) and ROI (34 ROIs) were set as fixed factors and subject as random factor. CT was specified as the dependent variable. In addition, effect sizes (Cohen's d) and percentage differences were calculated for each ROI.

#### Comparison of Healthy Controls and AD Patients Using FS6 and CAT12

Here, two linear mixed models were separately calculated for FS6 and CAT12 comparing HCs and AD patients to assess putative superiority of one method over the other in detecting brain atrophy in distinct brain regions. Group (HC, AD) and ROI (34 ROIs) were set as fixed factors, subject as random factor and total intracranial volume (TIV) was specified as covariate. CT was set as the dependent variable. In addition, effect sizes were calculated for each ROI.

#### Test‐Retest Analysis of FS6 and CAT12

To assess the reliability of both methods, a test‐retest analysis was carried out using the longitudinal processing pipelines implemented in both software packages. Test‐retest variability was calculated using the formula:

$$\%\mathrm{TRV}\;=\frac{\left|\mathrm{TP}1-\mathrm{TP}2\right|}{\left(\mathrm{TP}1+\mathrm{TP}2\right)/2}\;*100,$$

where time point 1 (TP1) indicates the first and time point 2 (TP2) the second scan.^23^ The median, the 25th, and 75th percentiles are reported for each region for both methods.^24^ Furthermore, Scatter‐ and Bland‐Altman plots were used for graphical representation to depict the degree of agreement for the respective data.^25^

## Results

**Figure 2:**
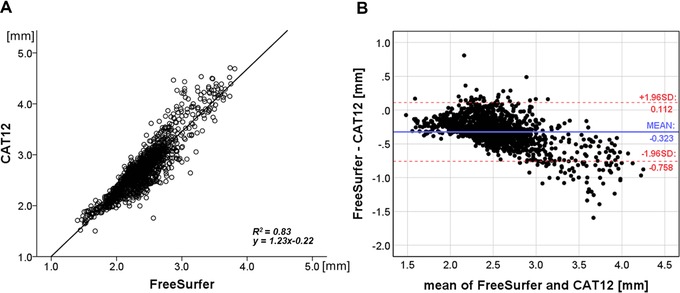
(A) Correlational analysis between FreeSurfer and CAT12 mean cortical thickness (CT) values. Each data point represents a CT value for a subject at a specific region. (B) Bland‐Altman plot with limits of agreement (dotted lines) for mean CT values indicating the agreement between both methods. *R* ^2^ = coefficient of determination; SD = standard deviation.

Independent‐samples student's *t*‐test showed no differences regarding age between the HC and the AD cohort (*t* = .119, *P* = .91). The correlational analysis between mean CT values of the HCs along all 34 ROIs between FS6 and CAT12 revealed a high coefficient of determination *R* ^2^ = .83 with the linear regression model *y* = 1.23*x*−.22. However, the Bland‐Altman plot suggested overall higher CT values for CAT12. These differences were even more pronounced at higher thickness values (Fig 2).

**Figure 3:**
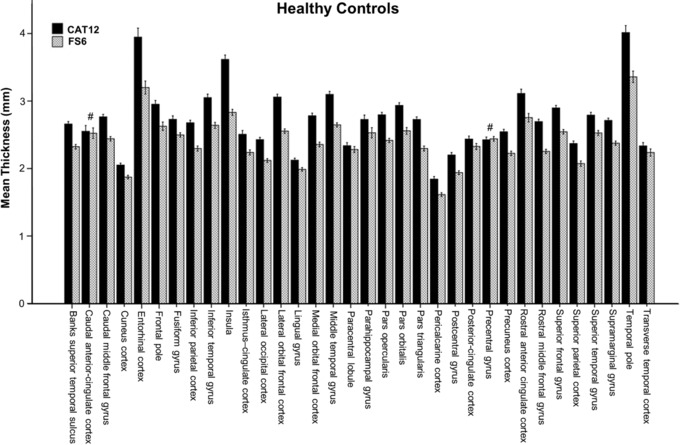
Mean cortical thickness values of FreeSurfer (FS6) and CAT12 for 34 regions of in interest (ROI). Thirty‐two of 34 ROIs showed significant differences (*P* < .05, corrected for multiple comparisons, nonsignificant results are marked with #). Error bars represent 95% CI.

To test whether these CT differences between the methods are significant, a subsequent mixed model analysis was conducted. According to Akaike's information criterion, the repeated covariance‐type “Toeplitz” was chosen. The analysis showed a significant interaction between method × ROI (*F*_33, 222.26_ = 56.378, *P* < .001). To interpret this interaction, post‐hoc paired *t*‐tests were carried out for each ROI between FS6 and CAT12 (Table 1, Fig 3). The CAT12 toolbox delivered significantly higher CT estimations in comparison to FreeSurfer in 32 of the 34 ROIs (*P* < .05, family‐wise error [FWE] corrected). No significant differences were found in the caudal anterior‐cingulate cortex and the precentral gyrus, while in the latter ROI, even higher CT values for FreeSurfer were observed. The most pronounced differences according to effect sizes were found for insula (Cohens’ *d* = 4.35, mean percentage difference Δ = 24.3%) lateral orbitofrontal cortex (*d* = 4.06, Δ = 18.0%), rostral middle frontal gyrus (*d* = 3.88, Δ = 17.8%), middle temporal gyrus (*d* = 3.54, Δ = 15.7%), medial orbital frontal cortex (*d* = 3.47, Δ = 16.5%), pars triangularis (*d* = 3.39, Δ = 17.1%), and pars opercularis (*d* = 3.36, Δ = 14.6%) (for detailed results, see Table 1).

**Table 1:** Comparison of Both Methods Including 44 Healthy Control Subjects

Region	FS6	CAT12	Δ (%) mean	Cohens' *d*	*R* ^2^
Banks superior temporal sulcus	2.32 ± .11	2.66 ± .12	13.4	2.86	.62
Caudal anterior‐cingulate cortex (n.s.)	2.52 ± .26	2.55 ± .28	6.4	.11	.43
Caudal middle frontal gyrus	2.44 ± .11	2.77 ± .12	12.5	2.76	.76
Cuneus cortex	1.87 ± .09	2.05 ± .10	9.1	1.97	.54
Entorhinal cortex	3.20 ± .32	3.95 ± .44	20.8	1.97	.34
Fusiform gyrus	2.50 ± .11	2.73 ± .17	9.0	1.65	.20
Inferior parietal cortex	2.30 ± .12	2.68 ± .12	15.3	3.09	.77
Inferior temporal gyrus	2.64 ± .14	3.05 ± .17	14.4	2.66	.49
Isthmus‐cingulate cortex	2.24 ± .13	2.51 ± .19	12.0	1.72	.18
Lateral occipital cortex	2.12 ± .09	2.43 ± .11	13.7	3.11	.23
Lateral orbital frontal cortex	2.55 ± .11	3.06 ± .13	18.0	4.06	.20
Lingual gyrus	1.99 ± .09	2.12 ± .10	6.7	1.44	.36
Medial orbital frontal cortex	2.36 ± .12	2.78 ± .13	16.5	3.47	.16
Middle temporal gyrus	2.65 ± .10	3.10 ± .15	15.7	3.54	.55
Parahippocampal gyrus	2.53 ± .25	2.73 ± .21	8.8	.85	.46
Paracentral lobule	2.28 ± .15	2.34 ± .15	4.2	.39	.57
Pars opercularis	2.42 ± .10	2.80 ± .12	14.6	3.36	.67
Pars orbitalis	2.56 ± .16	2.93 ± .14	13.8	2.49	.48
Pars triangularis	2.30 ± .12	2.72 ± .13	17.1	3.39	.71
Pericalcarine cortex	1.61 ± .09	1.84 ± .13	13.6	2.06	.40
Postcentral gyrus	1.94 ± .09	2.20 ± .12	12.5	2.41	.72
Posterior‐cingulate cortex	2.33 ± .15	2.44 ± .14	6.3	.76	.24
Precentral gyrus (n.s.)	2.44 ± .12	2.43 ± .14	3.1	–.12	.53
Precuneus cortex	2.22 ± .11	2.54 ± .12	13.4	2.85	.75
Rostral anterior cingulate cortex	2.75 ± .21	3.11 ± .22	12.4	1.70	.34
Rostral middle frontal gyrus	2.25 ± .10	2.69 ± .12	17.8	3.88	.76
Superior frontal gyrus	2.54 ± .11	2.90 ± .12	13.0	2.99	.84
Superior parietal cortex	2.07 ± .13	2.37 ± .12	13.4	2.40	.78
Superior temporal gyrus	2.53 ± .12	2.79 ± .14	9.9	2.00	.85
Supramarginal gyrus	2.38 ± .10	2.71 ± .12	13.2	3.09	.82
Frontal pole	2.63 ± .20	2.95 ± .19	12.0	1.66	.56
Temporal pole	3.36 ± .28	4.01 ± .35	17.7	2.07	.63
Transverse temporal cortex	2.24 ± .18	2.34 ± .16	6.2	.58	.47
Insula	2.83 ± .14	3.62 ± .22	24.3	4.35	.53

Mean cortical thickness values, standard deviations, and statistics for each region processed with FreeSurfer (FS6) and CAT12. 32 out of 34 regions showed significant higher estimates for CAT12.

Legend: n.s. = not significant; Δ (%) mean = mean percentage difference between both methods; *R* ^2^ = coefficient of determination.

To assess which method was more sensitive in detecting a statistical effect between the HCs and the AD cohort, two separate linear mixed models were carried out. Again, based on the Akaike's information criterion, the covariance‐type “Toeplitz” was chosen for both models in which CT estimations for FreeSurfer and CAT12 were investigated. The linear mixed model conducted for FreeSurfer, where the HC and AD cohorts are compared revealed a significant interaction between group × ROI (*F*_33, 263.03_ = 6.912, *P* < .001). The same analysis was carried out for the CAT12 toolbox, which also showed a significant interaction between group × ROI (*F*_33, 256.46_ = 11.805, *P* < .001).

As interactions were significant, post‐hoc group comparisons for CT corrected for TIV were conducted. Differences were statistically significant for 22 ROIs for FS6 and 25 for CAT12 (*P* < .05, FWE‐corr.).

**Figure 4:**
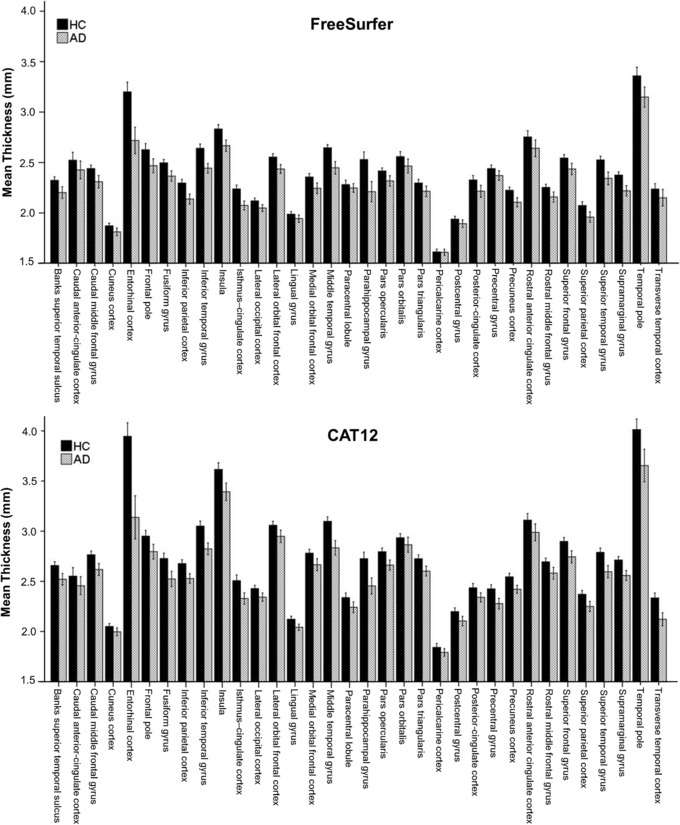
Mean cortical thickness (CT) values for FreeSurfer (FS6, upper panel) and CAT12 (lower panel) comparing healthy controls (HC, black) and an Alzheimer's cohort (AD, gray) using 34 regions of interest (ROIs). Both methods showed lower CT values for the AD cohort across the observed ROIs. These differences were statistically significant for 22 ROIs for FS6 and 25 for CAT12 (*P* < .05, corrected for multiple comparisons). For detailed results, see Table 2. Error bars represent 95% CI.

For graphical interpretation, see Figure 4. Most pronounced differences according to effect sizes between groups processed with both methods were found predominantly in temporal brain regions including the entorhinal cortex (FS6 *d* = 1.39, CAT12 *d* = 1.53), middle temporal gyrus (*d* = 1.40, 1.47), inferior temporal gyrus (*d* = 1.41, 1.36), supramarginal gyrus (*d* = 1.26, 1.18), superior temporal gyrus (*d* = 1.22, 1.21), and parahippocampal gyrus (*d* = 1.18, 1.22). (Detailed results can be found in Table 2.)

**Table 2:** Comparison of Healthy Controls and an Alzheimer's Cohort Using FreeSurfer (FS6) and CAT12

Region	FS6 HC	FS6 AD	CAT12 HC	CAT12 AD	FS6 Cohens' d	CAT12 Cohens' d
Banks superior temporal sulcus	2.32 ± .11	2.20 ± .17	2.66 ± .12	2.52 ± .17	.87*	.94*
Caudal anterior‐cingulate cortex	2.52 ± .26	2.43 ± .25	2.55 ± .28	2.46 ± .26	.38	.35
Caudal middle frontal gyrus	2.44 ± .11	2.31 ± .18	2.77 ± .12	2.62 ± .17	.91*	.99*
Cuneus cortex	1.87 ± .09	1.81 ± .11	2.05 ± .10	2.00 ± .11	.62	.52
Entorhinal cortex	3.20 ± .32	2.72 ± .38	3.95 ± .44	3.14 ± .62	1.39*	1.53*
Fusiform gyrus	2.50 ± .11	2.37 ± .15	2.73 ± .17	2.52 ± .22	1.02*	1.05*
Inferior parietal cortex	2.30 ± .12	2.14 ± .14	2.68 ± .12	2.53 ± .14	1.21*	1.13*
Inferior temporal gyrus	2.64 ± .14	2.44 ± .13	3.05 ± .17	2.82 ± .17	1.41*	1.36*
Isthmus‐cingulate cortex	2.24 ± .13	2.07 ± .13	2.51 ± .19	2.33 ± .16	1.29*	1.02*
Lateral occipital cortex	2.12 ± .09	2.05 ± .10	2.43 ± .11	2.34 ± .12	.75*	.75*
Lateral orbital frontal cortex	2.55 ± .11	2.44 ± .12	3.06 ± .13	2.95 ± .18	.99*	.70
Lingual gyrus	1.99 ± .09	1.94 ± .10	2.12 ± .10	2.04 ± .09	.45	.84*
Medial orbital frontal cortex	2.36 ± .12	2.24 ± .15	2.78 ± .13	2.67 ± .17	.83*	.77*
Middle temporal gyrus	2.65 ± .10	2.45 ± .18	3.10 ± .15	2.83 ± .21	1.40*	1.47*
Parahippocampal gyrus	2.53 ± .25	2.21 ± .29	2.73 ± .21	2.45 ± .23	1.18*	1.22*
Paracentral lobule	2.28 ± .15	2.25 ± .12	2.34 ± .15	2.24 ± .15	.25	.64
Pars opercularis	2.42 ± .10	2.32 ± .15	2.80 ± .12	2.66 ± .14	.79*	1.00*
Pars orbitalis	2.56 ± .16	2.47 ± .20	2.93 ± .14	2.86 ± .22	.51	.39
Pars triangularis	2.30 ± .12	2.21 ± .15	2.72 ± .13	2.60 ± .14	.60	.90*
Pericalcarine cortex	1.61 ± .09	1.61 ± .09	1.84 ± .13	1.79 ± .11	.04	.43
Postcentral gyrus	1.94 ± .09	1.89 ± .11	2.20 ± .12	2.11 ± .13	.46	.73
Posterior‐cingulate cortex	2.33 ± .15	2.22 ± .17	2.44 ± .14	2.34 ± .13	.70	.70
Precentral gyrus	2.44 ± .12	2.37 ± .14	2.43 ± .14	2.28 ± .15	.56	1.01*
Precuneus cortex	2.22 ± .11	2.11 ± .13	2.54 ± .12	2.42 ± .11	1.01*	1.06*
Rostral anterior cingulate cortex	2.75 ± .21	2.64 ± .24	3.11 ± .22	2.99 ± .24	.50	.53
Rostral middle frontal gyrus	2.25 ± .10	2.16 ± .14	2.69 ± .12	2.58 ± .17	.77*	.77*
Superior frontal gyrus	2.54 ± .11	2.44 ± .16	2.90 ± .12	2.74 ± .17	.80*	1.03*
Superior parietal cortex	2.07 ± .13	1.96 ± .15	2.37 ± .12	2.25 ± .15	.83*	.91*
Superior temporal gyrus	2.53 ± .12	2.34 ± .18	2.79 ± .14	2.60 ± .18	1.22*	1.21*
Supramarginal gyrus	2.38 ± .10	2.22 ± .15	2.71 ± .12	2.56 ± .14	1.26*	1.18*
Frontal pole	2.63 ± .20	2.47 ± .20	2.95 ± .19	2.80 ± .21	.79*	.77*
Temporal pole	3.36 ± .28	3.15 ± .29	4.01 ± .35	3.65 ± .47	.74*	.88*
Transverse temporal cortex	2.24 ± .18	2.15 ± .24	2.34 ± .16	2.12 ± .19	.42	1.22*
Insula	2.83 ± .14	2.67 ± .16	3.62 ± .22	3.39 ± .25	1.08*	.95*

Mean cortical thickness values, standard deviations, and effect sizes (Cohens’ *d*) for each region are shown. Values assigned with an asterisk indicate significant differences between the heatlhy controls (HC) and Alzheimer's cohort (AD) (corrected for multiple comparisons, *P* < .05).

### Test‐Retest Reliability

**Figure 5:**
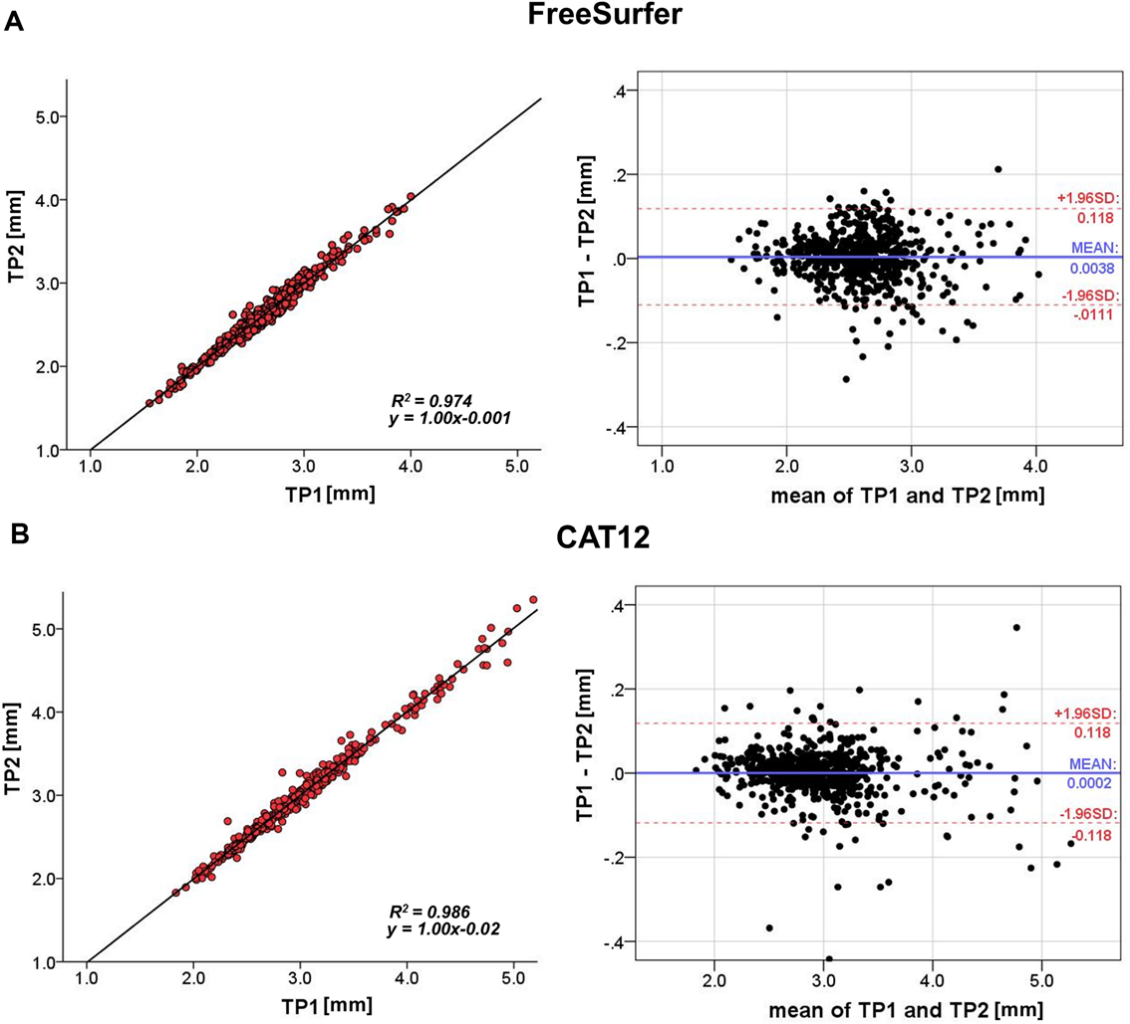
Test‐retest analysis showing correlational (left) and Bland‐Altman plots with limits of agreement (dotted lines, right) including 19 subjects and 34 regions for (A) FreeSurfer and (B) CAT12. TP = time point; *R* ^2^ = coefficient of determination; SD = standard deviation.

FS6 as well as CAT12 showed excellent test‐retest values with slightly better results for CAT12 (for detailed results per region, see Table 3). Similar *R* ^2^ values (FS6: *R* ^2^ = .974, *y* = 1.00*x* − .001; CAT12: *R* ^2^ = .986, *y* = 1.00*x* − .02) were observed (see Fig 5) when mean CT values from all subjects at each ROI were taken into consideration. Paired *t*‐tests between measurements from time point 1 and 2 showed no significant differences for FS6 (*t*‐val: 1.662, *P*‐val: .097) nor for CAT12 (*t*‐val: .097, *P*‐val: .923). Bland‐Altman plots showed a high level of agreement for both methods between the two time points (see Fig 5).

**Table 3:** Test‐Retest Performance of Both Methods

	FS6		CAT12		FS6 %TRV			CAT12 %TRV		
Region	M1	M2	M1	M2	median	25th	75th	median	25th	75th
Banks superior temporal sulcus	2.49 ± .13	2.50 ± .13	2.82 ± .13	2.82 ± .15	1.82	.69	2.61	.57	.37	1.09
Caudal anterior‐cingulate cortex	2.59 ± .15	2.60 ± .14	2.84 ± .17	2.84 ± .16	1.22	.62	2.37	.60	.33	.86
Caudal middle frontal gyrus	2.65 ± .08	2.65 ± .11	3.00 ± .11	3.00 ± .13	.90	.51	2.29	.67	.32	.90
Cuneus cortex	2.03 ± .13	2.03 ± .15	2.26 ± .15	2.25 ± .15	1.60	.66	2.10	1.09	.60	1.74
Entorhinal cortex	3.31 ± .27	3.35 ± .29	4.46 ± .41	4.49 ± .46	2.23	.93	3.28	1.18	.47	3.42
Fusiform gyrus	2.61 ± .09	2.63 ± .11	3.02 ± .13	3.02 ± .15	1.65	.47	2.53	.68	.44	1.31
Inferior parietal cortex	2.49 ± .09	2.48 ± .09	2.80 ± .09	2.81 ± .10	.71	.36	1.44	.65	.52	.86
Inferior temporal gyrus	2.76 ± .11	2.76 ± .14	3.23 ± .15	3.24 ± .14	1.19	.61	2.59	.67	.44	.98
Isthmus‐cingulate cortex	2.36 ± .16	2.35 ± .17	2.65 ± .18	2.62 ± .17	.89	.42	2.30	2.14	1.11	2.80
Lateral occipital cortex	2.25 ± .09	2.25 ± .11	2.53 ± .12	2.52 ± .12	1.16	.67	1.98	.68	.28	1.17
Lateral orbital frontal cortex	2.68 ± .12	2.68 ± .12	3.29 ± .14	3.27 ± .13	.94	.34	1.92	.94	.57	1.60
Lingual gyrus	2.18 ± .08	2.18 ± .10	2.42 ± .11	2.40 ± .13	1.13	.62	1.56	1.51	.45	2.24
Medial orbital frontal cortex	2.43 ± .15	2.44 ± .15	2.97 ± .16	2.95 ± .17	1.70	.50	2.68	1.03	.51	2.06
Middle temporal gyrus	2.82 ± .08	2.82 ± .11	3.39 ± .09	3.39 ± .11	1.29	.66	1.59	.45	.24	1.18
Parahippocampal gyrus	2.70 ± .19	2.69 ± .19	2.96 ± .16	2.96 ± .14	1.55	1.24	2.71	.91	.31	1.52
Paracentral lobule	2.49 ± .11	2.48 ± .12	2.66 ± .12	2.65 ± .13	1.04	.46	2.18	.76	.42	1.09
Pars opercularis	2.68 ± .08	2.68 ± .11	3.15 ± .11	3.15 ± .11	.99	.54	1.85	.83	.61	1.20
Pars orbitalis	2.76 ± .17	2.74 ± .19	3.25 ± .17	3.23 ± .19	2.08	.73	3.34	1.49	1.24	2.15
Pars triangularis	2.59 ± .09	2.57 ± .11	3.06 ± .12	3.05 ± .13	2.18	.60	3.22	.70	.41	1.04
Pericalcarine cortex	1.80 ± .10	1.80 ± .13	2.09 ± .10	2.08 ± .11	1.45	.44	3.60	1.62	.76	2.17
Postcentral gyrus	2.14 ± .12	2.13 ± .12	2.48 ± .12	2.49 ± .12	1.36	.53	1.57	.83	.48	1.32
Posterior‐cingulate cortex	2.45 ± .09	2.45 ± .10	2.60 ± .12	2.60 ± .12	1.28	.63	1.74	.58	.39	.82
Precentral gyrus	2.69 ± .10	2.68 ± .12	2.78 ± .11	2.78 ± .12	1.05	.34	1.91	.93	.61	1.24
Precuneus cortex	2.42 ± .10	2.42 ± .11	2.71 ± .11	2.70 ± .11	1.04	.32	1.74	.59	.32	1.26
Rostral anterior cingulate cortex	2.78 ± .21	2.81 ± .17	3.08 ± .27	3.15 ± .18	2.85	2.12	3.25	2.95	2.38	4.32
Rostral middle frontal gyrus	2.42 ± .09	2.42 ± .12	2.93 ± .12	2.92 ± .13	1.32	.67	1.69	.51	.22	.89
Superior frontal gyrus	2.81 ± .10	2.80 ± .13	3.23 ± .13	3.23 ± .14	1.39	.44	1.72	.25	.16	.61
Superior parietal cortex	2.25 ± .11	2.24 ± .12	2.54 ± .09	2.53 ± .10	1.42	.81	1.76	.23	.14	.71
Superior temporal gyrus	2.84 ± .08	2.82 ± .09	3.15 ± .13	3.17 ± .13	1.13	.87	1.99	.95	.37	1.85
Supramarginal gyrus	2.61 ± .10	2.60 ± .10	2.92 ± .10	2.92 ± .11	.96	.57	1.53	.76	.25	1.11
Frontal pole	2.84 ± .19	2.82 ± .23	3.20 ± .22	3.20 ± .24	1.97	.65	3.13	1.42	.97	2.19
Temporal pole	3.58 ± .22	3.57 ± .20	4.30 ± .30	4.28 ± .29	1.83	.63	2.21	1.26	.74	2.73
Transverse temporal cortex	2.67 ± .16	2.63 ± .18	2.91 ± .19	2.92 ± .16	2.58	.92	3.20	2.11	1.13	2.61
Insula	3.05 ± .11	3.06 ± .12	3.62 ± .32	3.67 ± .30	.81	.52	1.27	1.29	.95	2.63

Test‐retest metrics including two time points (M1 and M2) for FreeSurfer (FS6) and CAT12 are shown. Mean cortical thickness values, standard deviations, and statistics for test‐retest variability in percent (%TRV) are indicated for each region. Columns on the right show median, the 25th and the 75th percentiles of %TRV for FS6 and CAT12.

## Discussion

We compared ROI‐wise CT estimations of the surface‐based FS6 software and the volume‐based CAT12 toolbox for SPM using HCs, an Alzheimer's cohort for group comparison, and test‐retest data to assess reliability with scans from the OASIS database. CAT12 delivered significantly higher thickness estimations compared to FreeSurfer in almost all regions of the brain. These overestimations were highest in the insula with almost 25% percent difference between the two approaches. However, almost no differences were found for precentral gyrus and the caudal anterior‐cingulate cortex. Although overall higher CT estimations were evident for the CAT12 toolbox, a strong correlation was observed between both methods in terms of ROI mean CT estimations in the HC cohort, which indicates a systematic difference between the methods. However, as revealed by the Bland‐Altman plot, these differences were more pronounced at higher thickness estimations (>3 mm). Hence, CT estimations are not directly comparable in terms of absolute values, which must be considered when comparing studies analyzed with either of the two methods.

Subsequently, a group analysis was conducted to compare an Alzheimer's cohort to age and sex‐matched healthy subjects to assess whether both methods deliver comparable effect sizes in detecting brain atrophy in the form of lower CT estimates. As expected, FreeSurfer as well as CAT12 showed lower CT for the AD cohort compared to the HCs in all observed areas of the brain. In addition to the fact that CAT12 showed higher estimations for both cohorts when compared to FS6, results indicate similar effect sizes and most regions were statistically significant. However, the group × ROI interaction for CAT12 showed a higher *F*‐value, which may indicate better performance in detecting brain atrophy in the AD cohort.

The final test‐retest variability measurements yielded excellent values for both methods close to or even below 1, indicating that both methodological approaches provide robust assessments. However, CAT12 performed slightly better than FreeSurfer.

Interestingly, we found that the CAT12 toolbox delivered constantly higher CT values when compared to FS6 in almost all areas of the brain. As no in‐vivo gold standard is available, we cannot ultimately determine which of both methods is closer to the ground truth in terms of CT estimates. However, it has been shown that CT measures of FreeSurfer are in good agreement with postmortem data^1^ and histological observations.^26^ This was also corroborated by a study conducted by Rosas et al in which specific manually delineated cortical brain regions were compared to results gained via the standard FreeSurfer pipeline. Results showed no significant differences in CT metrics and manual assessments led to almost identical results.^27^ This evidence and the usage of FreeSurfer as a standard tool for cortical assessments suggests that CAT12 overestimates CT values. Nevertheless, as no full manual reconstruction of the entire cortex is available, we cannot assume that these prior evaluations of FreeSurfer data are valid for all cortical brain regions. The differences between both methods, however, might be explained by the completely different approaches for calculating CT. In line with this, it has already been discussed that different CT estimation approaches can lead to different results.^7^, ^28^ In the FreeSurfer pipeline, the WM and pial surfaces are reconstructed and the distance is calculated between these reconstructed surfaces at each location of the vertex.^1^, ^10^ On the other hand, CAT12 uses a volume‐based approach using PBT, where a total reconstruction of the surfaces is not necessary. A projection scheme is used, taking blurred sulci into consideration to calculate the central surface.^11^ It is possible that these blurred regions play a key role in the observed differences between the two methods.

Of note, one recently published study^29^ did not find CAT12 overestimations, in contrast to our results. Moreover, their results suggest that CAT12 CT estimations were lower compared to FreeSurfer with respect to several brain regions. These differences may be explained by use of a beta version of CAT12 (r720) with manually processed ROI extractions. Furthermore, FreeSurfer 5.3 was used. We used the latest available software versions and also processed the data according to standard procedures including the built‐in ROI extractions. Furthermore, we could demonstrate that this effect of CAT12 CT overestimations is age‐independent as it was observed for the older cohorts, as well as for our test‐retest group that consisted of healthy young adults (for more details, see Tables 1, 2, 3). Although 1.5 Tesla brain scans were used for our investigation, the scans of the OASIS data were of high quality, as subjects were recorded three to four times within one session and an average image has been constructed. To achieve better matching and a reduction in variance, only female subjects were included in our cross‐sectional analysis where Alzheimer's patients and HCs were compared. However, we assume generalizability of our results and do not expect gender‐related influences on CT measurements.

Our analyses provide reasonable evidence that the CAT12 toolbox delivers accurate and robust results and can be considered a fast, easy‐to‐use, and reliable alternative to FreeSurfer when processing resources are limited as no extensive cortical reconstruction steps are needed. Reduced processing time is of particular benefit in contrast to FreeSurfer. While preprocessing in CAT12 can be conducted within 1 hour per subject, the processing in FreeSurfer takes about 10–20 hours as the entire surface is reconstructed. In addition, the fixing of putative topological defects can lead to even longer processing times. However, CAT12 showed higher thickness estimations in almost all investigated brain regions compared to FreeSurfer. Hence, future studies using CAT12 must keep these overestimations in mind, especially when results are compared to other CT studies.
